# Anatomical variations of the palmaris longus muscle including its relation to the median nerve – a proposal for a new classification

**DOI:** 10.1186/s12891-017-1901-x

**Published:** 2017-12-19

**Authors:** Łukasz Olewnik, Grzegorz Wysiadecki, Michał Polguj, Michał Podgórski, Hubert Jezierski, Mirosław Topol

**Affiliations:** 1 Department of Normal and Clinical Anatomy, Interfaculty Chair of Anatomy and Histology, Medical University of Lodz, ul. Narutowicza 60, 90-136 Łódź, Poland; 2 Polish Mother’s Memorial Hospital Research Institute, Lodz, Poland; 3 Department of Angiology, Interfaculty Chair of Anatomy and Histology, Medical University of Lodz, Lodz, Poland; 4 Department of Trauma and Orthopaedic Surgery, Hospital of Ministry of Interior and Administration in Lodz, ul. Północna 42, 91-425 Łódź, Poland

**Keywords:** Palmaris longus muscle, Palmaris longus tendon, Tendon grafts, New classification

## Abstract

**Background:**

The palmaris longus (PL) muscle is characterised by high morphological diversity, and its tendon crosses the median nerve (MN) at different levels. Due to the fact that the palmaris longus tendon is routinely harvested for reconstruction of other tendons, knowledge of its morphological variations is clinically important. Therefore, the purpose of the study was to suggest a new morphological classification of the PL muscle and characterise the relationship of its tendon to the median nerve.

**Methods:**

Standard dissection was performed on 80 randomised and isolated upper limbs (40 left and 40 right) fixed in a 10% formalin solution. Measurements of muscle belly and tendon were obtained. The course and location of tendon insertion, as well as its relationship to the median nerve, were noted.

**Results:**

The palmaris longus muscle was present in 92.5% of specimens. Three types of palmaris longus muscle were identified based on the morphology of its insertion (types I-III) and these were further subdivided into three subgroups (A-C) according to the ratio of the length of the muscle belly and its tendon. The most frequent was type I (78.8%), where the tendon attached to the palmar aponeurosis, and subtype B, where the tendon-to-belly ratio was 1–1.5 (41.1%). The mean distance from the interstyloid line to the crossing between the median nerve and the palmaris longus tendon was 31.6 mm. In addition, two types of palmaris longus were described.

**Conclusion:**

The presented classification of palmaris longus muscle types allows a better characterization of its diversity and may be useful in planning tendon grafting.

## Background

The palmaris longus (PL) is a narrow, fusiform muscle of the superficial anterior compartment of the forearm [1–6]. The muscle originates at the medial epicondyle of the humerus and the antebrachial fascia, with the muscle belly partially fused with the adjacent muscles [3, 4]. The muscle belly turns into the long tendon and inserts in the palmar aponeurosis [2–4]. The PL muscle is located between the flexor carpi radialis and the pronator teres muscles [3, 5]. Its course partly overlaps the median nerve [2, 3].

The palmaris longus muscle, just like the plantaris muscle, presents morphological variability [7, 8]. Variations in PL morphology are not uncommon: they may include an additional belly of the muscle, fusion with another muscle, bifurcated tendon, reversed muscle, an atypical tendon course and inserts, or multiple tendinous insertion [2, 6, 9–12]. A knowledge of PL variations is important due to its role in vascular-neural compression [3, 13, 14], and its use as a graft for tendon/ligament reconstruction [2, 5, 6, 9, 11, 12, 15, 16]; especially in reconstructive hand surgery [2, 5, 6, 9, 11, 12, 15–17]. It is most frequently used as a graft for the finger flexors [15, 17]. The PL muscle tendon is a perfect choice for grafts, because it meets the most important criteria, such as the necessary length and diameter, and the functionality of the upper limb is undisturbed by the harvesting of the PL tendon. Furthermore, as the myofascial relationships between the muscles, tendons and adjacent structures may play an important role in understanding both functional nuances and background of specific clinical symptoms [18], the myofascial relationships of PL may play a role in the pathogenesis of the carpal tunnel syndrome [18]. The previous classifications were anatomical; however, as the PL tendon is so suitable for transplantation, due to its length, an accurate classification should be introduced which will take into account the tendon-to-belly ratio and great morphological variability. The strength of our proposed classification is that is has these two features and also describes the relationship between the palmaris tendon and median nerve.

The purpose of this study is to suggest a new classification of the palmaris longus based on its morphological variability, and to determine the relationship of the muscle to the median nerve as well as antebrachial fascia, flexor retinaculum and palmar aponeurosis. In our opinion, the new classification will be of great value in planning the transplantation of this tendon.

## Methods

Eighty randomly-selected isolated (40 right and 40 left) upper limbs were obtained from adult cadavers, and fixed in 10% formalin solution before examination.

The Local Bioethics Commission issued a consent for the study (agreement no. RNN/92/16/KE).

A dissection of the forearm and hand area was performed by traditional techniques [7, 8, 19, 20]. Upon dissection, the morphology of the palmaris longus was assessed, together with the location and type of its insertion to the palmar aponeurosis.

The next stage comprised a set of morphometric and anthropometric measurements of the belly and tendon of the palmaris longus muscle. The distance from the midpoint of the interstyloid line (a line drawn between the styloid processes of the radius and the ulna) and the crossing point the median nerve and the PL muscle tendon were assessed. An electronic digital calliper was used for all measurements (Mitutoyo Corporation, Kawasaki-shi, Kanagawa, Japan). Each measurement was carried out twice with an accuracy of up to 0.1 mm. All morphometric measurements were subjected to statistical analysis.

The tendon-to-muscle ratio, defined as the relationship between the length of the tendon and the length of the muscle belly, was calculated. Based on this coefficient, types I-II were subdivided into subtypes A (tendon-to muscle-ratio < 1), B (tendon-to muscle-ratio 1–1.5) or C (tendon-to muscle-ratio > 1.5).

The statistical analysis was performed using Statistica 12 software (StatSoft Polska, Cracow, Poland). A *p*-value below 0.05 was considered significant. The results are presented as a mean and standard deviation unless otherwise stated.

The normality of the continuous data distribution was checked with the Shapiro-Wilk test. As the data was not normally distributed, the Mann-Whitney test was used to compare the anthropometric measurements between two types of the palmaris longus muscle. The correlation of continuous variables was assessed with the Spearman’s rank correlation coefficient.

## Results

Three types of palmaris longus muscle were recognised based on variations in its insertion (Types I, II, III) – Table 1. Type I and Type II were further divided into subtypes A,B and C based on variations in the tendon-to-muscle length ratio (Table 2). All types originated on the medial epicondyle of the humerus.

**Table 1:** Classification of Types and Subtypes of the palmaris longus muscle

Type	Inclusion criteria for the type	Subtype	Inclusion criteria for the subtype
Type I	• origin of the muscular part of the medial epicondyle of the humerus • the tendon was inserted to the palmar aponeurosis	A	Tendon to muscle ratio < 1
		B	Tendon to muscle ratio 1–1,5
		C	Tendon to muscle ratio > 1,5
Type II	• origin of the muscular part of the medial epicondyle of the humerus • the tendon was bifurcated (lateral and medial division); the lateral division was inserted to the palmar aponeurosis, and medial division was inserted to the flexor retinaculuum of the wrist	A	Tendon to muscle ratio < 1
		B	Tendon to muscle ratio 1–1,5
		C	Tendon to muscle ratio > 1,5
Type III	“Rare variants” non-conforming definition Types I and II

**Table 2:** Distribution of types and subtypes of the palmaris longus tendon

Palmaris longus tendon types	Palmaris longus tendon subtype [n (%)]		
	A	B	C
I (*n* = 63)	12 (19.1)	27 (42.9)	24 (38.1)
II (*n* = 10)	3 (30.0)	3 (30.0)	4 (40.0)

**Fig. 1:**
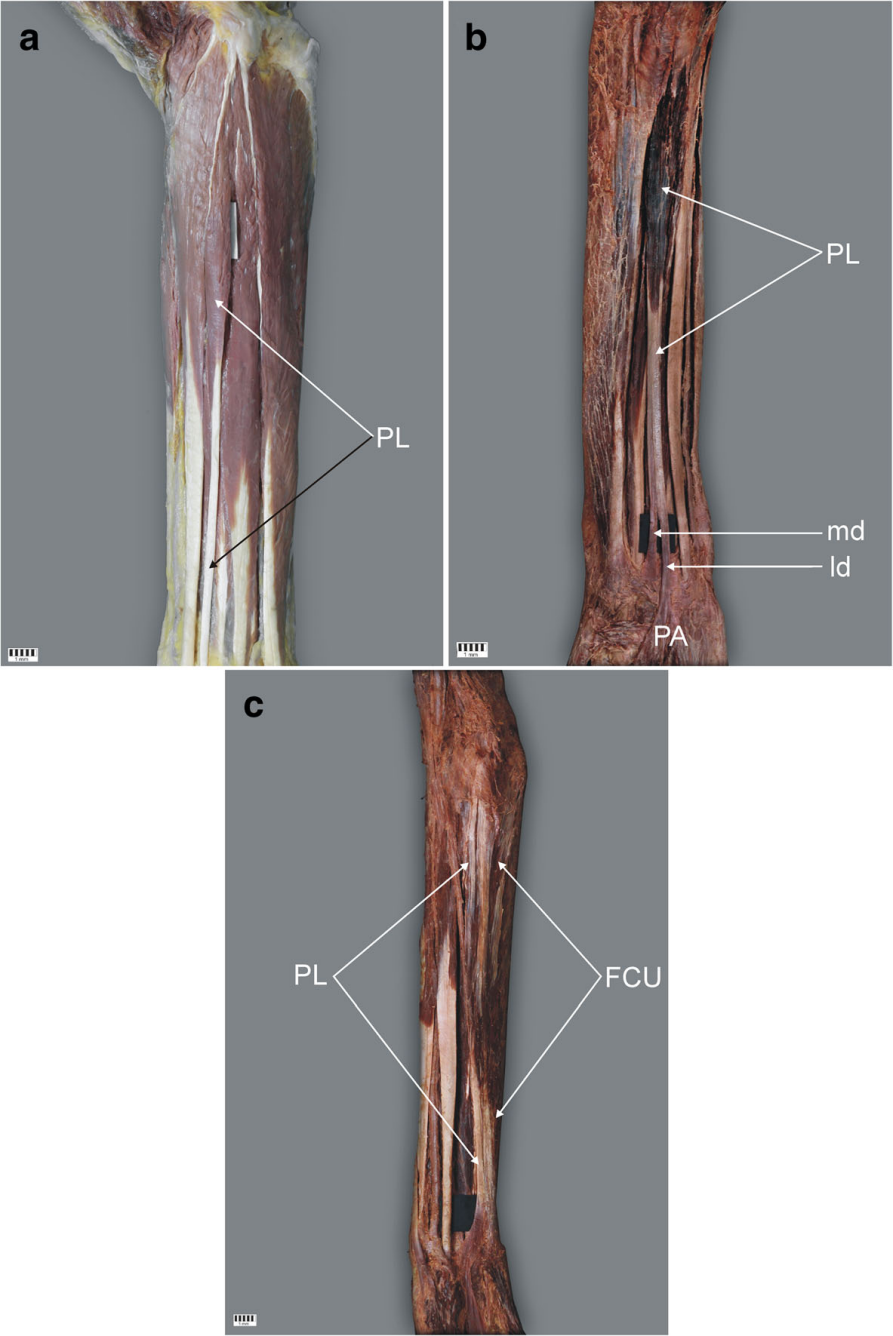
Palmaris longus muscle. **a** Type I of the palmaris longus muscle. Right forearm. **b** Type II of the palmaris longus muscle. Left forearm. **c** Type III of the palmaris longus muscle. Right forearm. *PL* palmaris longus muscle, *PA* palmar aponeurosis, *FCU* flexor carpi radialis muscle, *md* medial division of the tendon of palmaris longus muscle, *ld* lateral division of the tendon of palmaris longus muscle

**Fig. 2:**
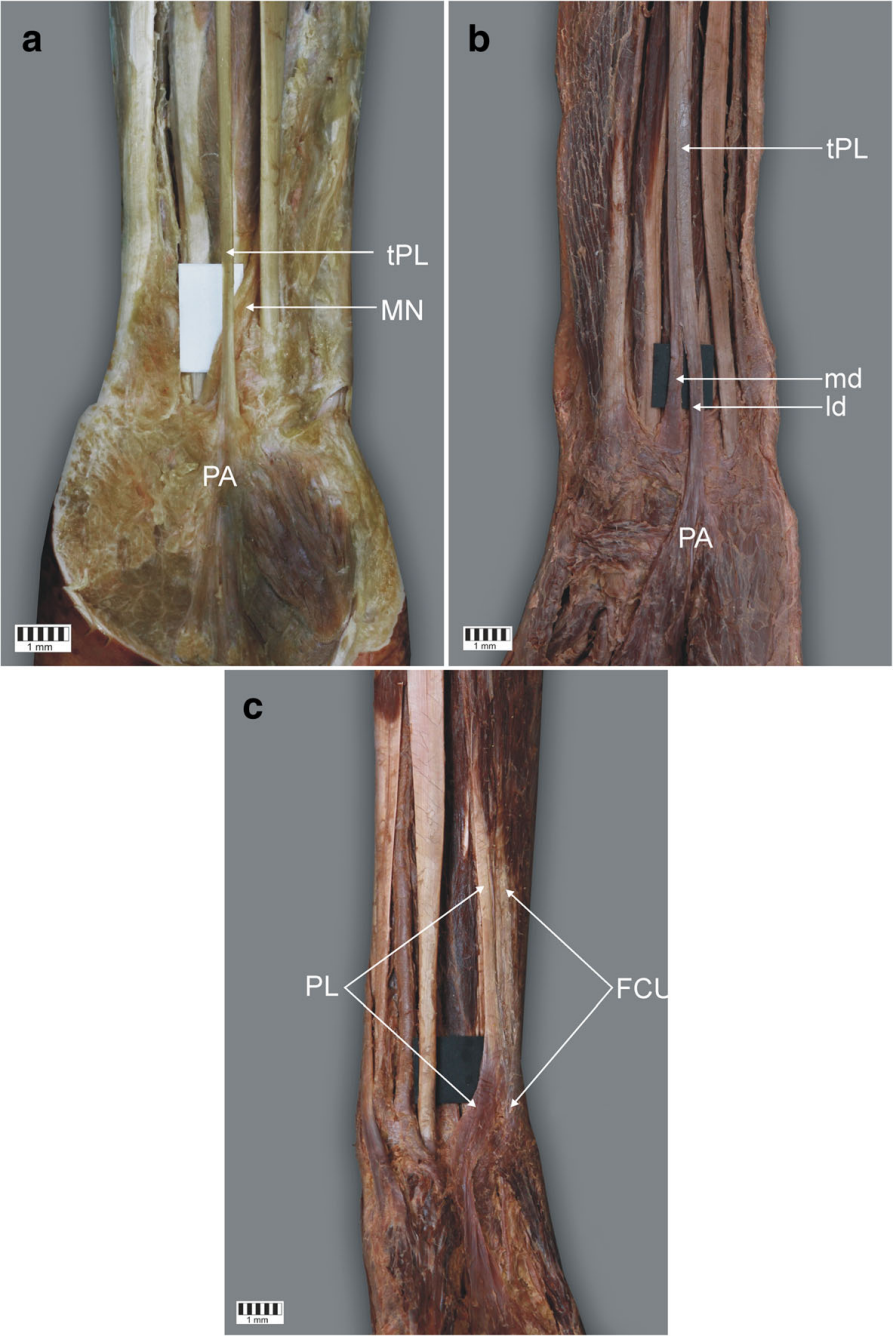
Insertion of the tendon of palmaris longus muscle. **a** Insertion of Type I of the palmaris longus muscle. Left forearm. **b** insertion of type II of the palmaris longus muscle. Left forearm. **c** Insertion of type III of the palmaris longus muscle. Right forearm. *tPL* tendon palmaris longus muscle, *MN* Median nerve, *PA* palmar aponeurosis, *md* medial division of the tendon of palmaris longus muscle, *ld* lateral division of the tendon of palmaris longus muscle, *FCU* Flexor carpii radialis

Type I was characterised by the origin of the muscular part of the medial epicondyle of the humerus, with the muscle belly turned into the tendon and the insertion located on the palmar aponeurosis. This type was observed in 63 upper limbs (78.8%) [Figs. 1a, 2a].

Type II demonstrated a proximal attachment with the same morphology as Type I. However, the tendon was bifurcated: the lateral division of the tendon always predominated and was inserted in the palmar aponeurosis, the mean length of the tendon being 39.7 mm, while the medial division of the tendon was auxiliary and inserted in the flexor retinaculum of the wrist (mean length 26.5 mm). The mean distance from the interstyloid line, between the styloid processes, and the tendon bifurcation point was 32.9 mm. This type was found in 10 upper limbs (12.5%) [Figs. 1b, 2b].

Type III was categorised as ‘rare variations’. Namely, in one limb, the palmaris longus muscle was observed to be fused with the flexor carpi ulnaris muscle. In the distal part of the tendon, the insertion is visible in the palmar aponeurosis and in the pisiform bone. This type was found in only 1.2% of cases [Figs. 1c, 2c].

The Type I tendon predominated throughout the whole group (63 cases, 78.8%). Type II was found in 10 cases (12.5%). The coefficient lengths of the palmaris tendon and the belly ranged from 0.50 to 2.32 (mean 1.4 ± 0.36). The distribution of the types and subtypes of the palmaris longus tendon is presented in Table 2.

**Fig. 3:**
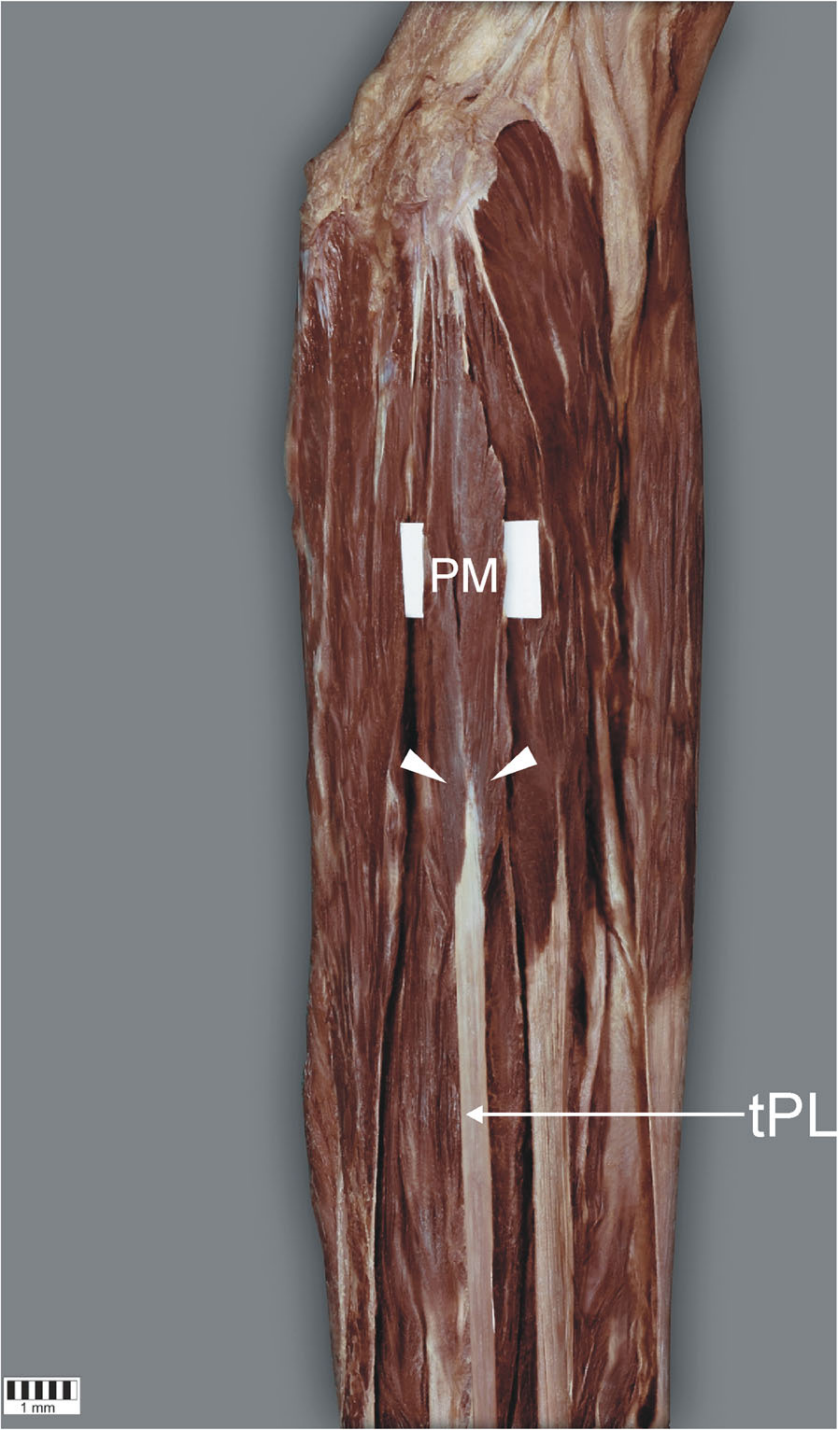
Muscle with low pinnate. The white arrowheads indicate the pennation. *PM* palmaris longus muscle, *tPL* tendon palmaris longus muscle

In the Type I group, low bipennation of the muscle was found in five cases (6.25%) [Fig. 3], while the tendon was located adjacent to the muscle belly in the other 75 cases (93.75%). In all cases, the muscle belly was located deep to the antebrachial fascia at the site where the bicipital aponeurosis blends in the antebrachial fascia.

The dimensions of the palmaris longus tendon and the median nerve at the point of their crossing are presented in Table 3. The nerve was significantly thicker and wider than the tendon. However in 15% of cases, the tendon was wider than the nerve, and in 8%, the structure was thicker. Moreover, there was a significant positive correlation between both thickness and width of the tendon and the nerve (R^2^ = 0.43, *p* = 0.029 and R^2^ = 0.41, *p* = 0.038 respectively).

**Table 3:** Dimensions of the median nerve and tendon of palmaris longus muscle where they cross

Dimension of structure		Mean [mm]	SD [mm]	p-value
Thickness	Tendon	1.14	0.43	0.0056
	Nerve	1.94	0.55	
Width	Tendon	3.82	0.96	0.0000
	Nerve	4.54	1.23	

**Fig. 4:**
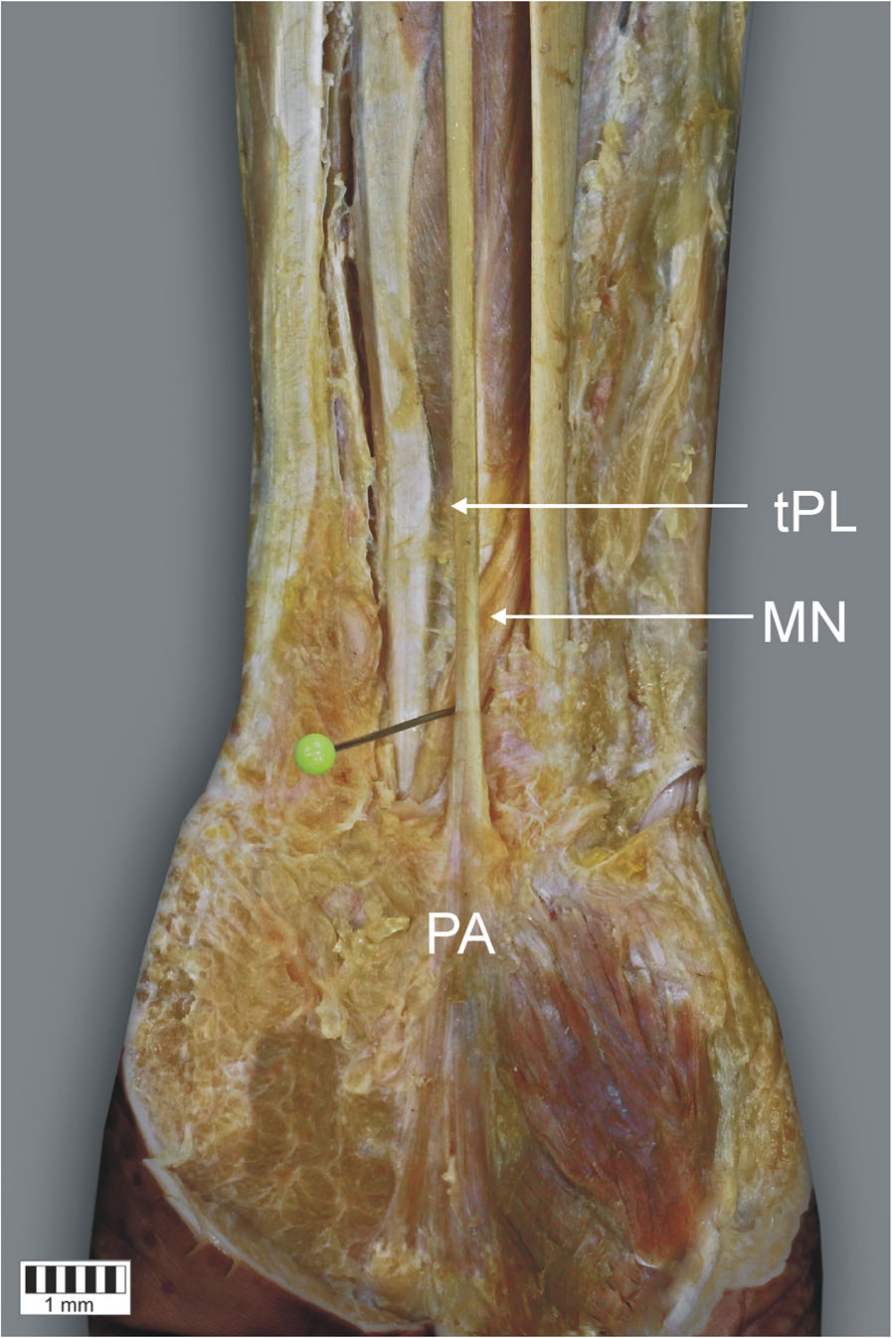
Relationship between median nerve (*MN*) and the tendon of the palmaris longus muscle (*tPL*)

The mean distance from the midpoint of the interstyloid line to the point where the median nerve crosses the palmaris longus tendon was 31.6 (SD = 7.2) mm. The typical relationship between the course of the palmaris longus muscle and the median nerve is presented in Fig. 4. There was no significant difference between tendon types with regard the mean distance from the interstyloid line to the crossing of the tendon and the median nerve (*p* = 0.4480). However, in Type II, the tendon split in the region where it crossed the median nerve. The mean distance from the interstyloid line to the crossing and to the split were 31.6 (SD = 7.2) and 32.9 mm (SD = 1.4), respectively (*p* = 0.5621).

The comparison of morphometric measurements between two types of the palmaris longus muscle tendon shows that only the width of the muscle tendon in its distal portion was significantly lower in Type II than Type I (Table 4). The palmaris longus muscle was found to be absent in six limbs: two left and four right ones (7.5% cases).

**Table 4:** Comparison of measurements between types I and II of the palmaris longus muscle

Morphometric parameter	Type of the palmaris longus		*p*-value
	Type I	Type II	
Forearm length [mm]	254.8 (24.3)	252.0 (13.6)	0.7181
Forearm circumference [mm]	212.5 (24.8)	217.2 (22.0)	0.5107
Width between processes	55.4 (4.2)	54.1 (4.0)	0.2788
Thickness between processes	163.9 (21.4)	170.2 (15.5)	0.1883
The length of the belly	115.0 (21.6)	107.4 (19.5)	0.1510
The length of the tendon	155.9 (29.4)	140.0 (32.7)	0.2614
The thickness of the tendon at the ‘muscle-tendon’ junction	1.7 (0.7)	2.2 (0.7)	0.0831
The width of the tendon at the ‘muscle-tendon’ junction	3.7 (1.6)	4.0 (1.7)	0.9361
The thickness of the tendon at the insertion point	2.1 (1.0)	1.6 (0.4)	0.0905
The width of the tendon at the insertion point	4.4 (1.4)	3.0 (1.0)	0.0018
The width of the central portion of the tendon	4.0 (1.2)	3.4 (1.2)	0.1216
The width of the tendon where the tendon crosses the median nerve	3.9 (0.9)	3.4 (1.7)	0.4222
The thickness of the tendon where the tendon crosses the median nerve	1.2 (0.4)	1.1 (0.2)	0.7788
Distance from the midportion of the interstyloid line to the point where the median nerve crosses the tendon	32.0 (7.4)	28.5 (5.8)	0.3773

## Discussion

The palmaris longus is one of the most variable muscles in the human body [3, 4], and one which demonstrates morphological variability in both the muscle belly and its tendon. The descriptions found in the literature are mainly focused on the morphology of the muscle [3, 4, 9, 10, 12–17, 21, 22]. Although a classification of muscle structure types based on the ratio between the length of the belly and that of the tendon may certainly be useful in procedures based on palmaris tendon grafts, no such classification currently exists.

Possibly the first description of the anatomical variations of the PL muscle was given by Anson et al. [19]. Their study on 1600 limbs found the PL to be absent in 205. The incidence of anomalies of all types, eclusive of agnesis, was 46 in 530 consecutive arms. One half of these anomalies (23 in 46) comprised variations in position and form, while the remainder comprised accessory slips and substitute structures (15 examples), duplication of the PL (four times) and aberrancies of attachment (three times) [19].

In a study performed on 48 upper limbs, Mathew et al. [3] classify samples as normal morphology, complete agenesis and other morphological types [3]. The most common type was ‘normal palmaris longus’, characterised by an origin located on the medial epicondyle and an insertion to the palmar aponeurosis: This type was found in 39 upper limbs (81.25%) [3]. The corresponding type in the present study (Type I) was found in 63 limbs (78.8%).

Among other varieties of palmaris longus, Mathew et al. [3] identify reverse palmaris longus, characterised by a tendinous origin located on the epicondyle of the humerus, with the origin of the muscle belly on the lower 2/3 of the forearm and inserted to the flexor retinaculum and the pisiform bone [3]; this type was only identified in one limb (2.08%). They also report the occurrence of an accessory PL in one limb (2.08%) and a ‘fleshy’ type in another (2.08%), the latter being characterised by apprearing broad, fleshy and bipennate in its upper half but unipennate in the distal third, with fibres fanning out from the radial side all the way to its insertion. They also note a fourth or fusifom type of palmaris longus, characterised by a belly occupying the central one-third, which is originated and inserted by means of thin tendons in the upper and lower thirds, respectively. This type deprives the subject of a source of a lengthy tendon (1 case – 2.08%) [3].

None of the above types were found in the present study. However, their final ‘rare variation’ is similar to our Type II, labelled the ‘bifurcated tendon’; it is characterised by a bifurcation of the tendon into medial and lateral parts with the medial part inserted in the palmar aponeurosis and the lateral part in the flexor retinaculum of the wrist (one case – 2.08%) [3]. This type was identified in 10 limbs in the present study as Type II (12.5%).

Some variants of the palmaris longus muscle have also been described as case reports. Bernardes et al. [12] describe a combined variation of palmaris longus and flexor digitorum superficialis: in this case, the palmaris longus tendon was found to pass beneath the flexor retinaculum inserted at the base of the middle phalanx of the fourth digit and replace the tendon of the flexor digitorum superficilis [12]. Marpalli et al. [6] and Murabit et al. [9] have reported the presence of the reverse palmaris longus muscle as case studies. Igbal et al. [11] describe the occurrence of a bitendinous PL muscle, while Barkats [10] describe a case of hypertrophy of the palmaris longus muscle as a rare anatomical variation. Kumar et al. [2] identify a rare variant of the PL with multiple tendinous insertions.

In our opinion, the case reports given above, including those described by Mathew et al. [3] as other morphological types (except the ‘bifurcated tendon’ type) should be classified as rare variants of the PL muscle and included into the Type III classification proposed in this paper. Every other morphological type described as a case report or differing from Types I and II might also be included in this group.

The PL was found to be absent in six limbs examined in the present study (7.5% cases). Similarly, Mathew [3] report the absence of a PL in four limbs (8.35%). Numerous other papers refer to the frequency of occurrence of this muscle in specific populations. Namely, Ceyhan et al. [21] found this muscle to be absent in 63.9% of a studied Turkish population, and another study reports a very high absence in an Egyptian population (50.8%) [23]. In contrast, the muscle was found to be absent in only 1.5% of limbs in a Zimbabwean population [24], and 3.1% - 3.8% in a Ghanaian population [25]. Sebastin et al. [26] describe a lack of the palmaris longus muscle in 4.6% limbs in an Asian population.

Attention should be drawn to the specific fascial relationships between the PL and the antebrachial fascia as well as the palmar aponeurosis. Typically, the muscle is situated deep inside the antebrachial fascia. Its tendon, however, moves to a suprafascial plane in the lower third of the forearm and is in continuity with the longitudinal fibers of the palmar aponeurosis [18]. According to Stecco et al. [18] the PL should be considered as a tensor of the superficial fascial system of the subcutaneous tissue. Occasionally, the PL tendon may shorten and become calcific and brittle with age, resulting in discomfort and even pain in the palm of the hand. According to Keese et al. [1] the PL tendon may play an important role in the pathophysiology of carpal tunnel syndrome and is a strong independent risk factor for carpal tunnel syndrome. Moreover, Keese et al. [1] report that “the prevalence of palmaris longus agenesis was significantly lower in the carpal tunnel group”. The counterpart of the PL in the leg is the plantaris muscle, and the plantaris muscle may also play an important role in Achilles, plantaris, or calf pain syndromes [7, 8], Smith et al. [27] emphasize the need for a sonographic classification system for plantaris tendon anatomy and motion with the goal of differentiating normal from pathologic states, and identifying risk factors for symptom development. Similar recommendations may be applied to the anatomy and motion of the PL tendon – especially with regard to age and eventual presence of the clinical symptoms.

Due to the fact that the PL muscle tendon is primarily used for grafts, Cetin et al. [22] found that people with the absence of the muscle reported no ailments related to their daily activities. Therefore, the graft of this tendon should presumably have no effect on the functionality of the hand [17]. Our proposed division of types I and II into subtypes will be of value while making decisions regarding the grafting of this tendon. The tendon tended to be longer in Type I muscles, which suggests that this type may be more appropriate for tendon transfer. This hypothesis is supported by the fact that the Type II tendon is bifurcated: when harvesting there is a risk that only one strip will be grasped and the tendon will be torn. Moreover the place where the tear may occur (region of tendon splitting) overlies the location where it crosses the median nerve. Therefore, an increased risk of nerve injury with the harvesting loop is associated with tearing the tendon. Proper tendon ablation is crucial in surgical procedures. The tendon ablation starts with locating the tendon at the proximal wrist crease and making the incision; incisions are then made every 5 cm. From these short cuts, the distal tendon is removed and is pulled under the skin in the proximal direction [28]. In addition, the PL tendon is an important landmark for palm access for treating carpal tunnel syndrome, distal radial fracture and Guyon channel syndrome [1, 3, 26].

The median nerve might be also injured due to other causes. Although no symptoms are typically associated with the anatomic variants of the palmaris longus muscle, there are some reports which suggest that the presence of the reverse palmaris muscle may cause compression on the median nerve [13, 14, 29–31]. Moreover, while our findings indicate that the median nerve was larger than the tendon at the point of their crossing in the majority of cases, this was not the case in 8–15% of specimens; in such cases, the size of the structure might be misleading and the median nerve may be potentially mistaken for a PL muscle tendon while harvesting [32].

Therefore, a detailed evaluation of the tendon-nerve crossing point is crucial when performing such surgical procedures as median nerve repair or tendon harvesting.

## Conclusion

The proposed classification of the PL muscle presented above is a potentially significant one because the diversity of morphological variants of both the muscle belly and the insertion of PL is an important consideration when performing palmaris longus graft surgeries.

In addition, surgical procedures performed in this region require accurate knowledge of the mean distance between the interstyloid line and the crossing of the median nerve with the palmaris longus tendon.

## Abbreviations

MN: Median nerve
PL: Palmaris longus
